# Analysis of *Lactobacillus rhamnosus* GG in Mulberry Galacto-Oligosaccharide Medium by Comparative Transcriptomics and Metabolomics

**DOI:** 10.3389/fnut.2022.853271

**Published:** 2022-03-18

**Authors:** Erna Li, Qiaoling Zhu, Daorui Pang, Fan Liu, Sentai Liao, Yuxiao Zou

**Affiliations:** Key Laboratory of Functional Foods, Ministry of Agriculture and Rural Affairs, Guangdong Key Laboratory of Agricultural Products Processing, Sericultural and Agri-Food Research Institute, Guangdong Academy of Agricultural Sciences, Guangzhou, China

**Keywords:** *Lactobacillus rhamnosus* GG, mulberry galacto-oligosaccharide, prebiotics, transcriptome analysis, metabolome analysis

## Abstract

*Lactobacillus rhamnosus* GG (LGG) has strong acid resistance and can survive passing through the stomach to colonize the intestines, where it promotes the growth of beneficial bacteria. Prebiotics such as mulberry galacto-oligosaccharide (MGO), mulberry polysaccharide solution (MPS), and galactooligosaccharides (GOS) promote LGG proliferation, and MGO has the greatest effect. After culturing LGG with prebiotics, changes in gene expression were studied at the transcriptomic and metabolomic levels. The results showed that, in the stable 24-h growth period of cultivation, ~63 and 132% more differential genes were found after MPS and MGO were added to the MRS medium, respectively, than after GOS was added, and the numbers of up-regulated genes were about 18 and 66% higher with MPS and MGO, respectively, than GOS. Analysis using the KEGG database revealed that, when LGG was cultured with MGO, 120 genes that were up-regulated as the growth rate increased were mainly enriched in pathways such as membrane transport, amino acid metabolism, and carbohydrate metabolism. The genes *gatB* and *gatC* were up-regulated for galactose metabolism, and *bglA* was up-regulated in the glycolysis/gluconeogenesis pathway. The qRT-RCR results, which were in agreement with the RNA-seq, indicated the genes involved in the proliferation effect of LGG were up-regulated. UDP-glucose may be a key metabolite for MGO to promote LGG proliferation.

## Introduction

Probiotics that can promote the proliferation of lactic acid bacteria have been reported in many research articles. Isomaltooligosaccharide (1), inulin (2), galactooligosaccharides (3), and fructooligosaccharide (4) have all been demonstrated to promote the growth of lactic acid bacteria and bifidobacteria. These oligosaccharides have high stability and are not easily decomposed by enzymes in the digestive tract and reach the intestine directly. They provide nutrients for beneficial bacteria, such as *Lactobacillus* and *Bifidobacterium*, and promote their proliferation but cannot be used by harmful bacteria. Therefore, selective proliferation by these probiotics can maintain the microecological balance of the digestive tract and benefit human health. Different types of prebiotics can promote the proliferation of probiotics. Huebner et al. (5) found that commercially produced fructooligosaccharide (FOS), galactooligosaccharides (GOS), and inulin had a proliferative effect on five *Lactobacilli* and five *Bifidobacterium*. Among them, inulin had the most significant effect on *Lactobacillus casei* 1195. Compared with traditional commercial prebiotics, polysaccharides extracted from natural plants also show good prebiotic activity. Azmi et al. (6) studied the *in vitro* proliferative effect of bamboo shoot crude polysaccharides on *Bifidobacterium* and *L. acidophilus* and found it to be better than that of FOS. Crude bamboo shoot polysaccharide has an average molecular weight of 7,000 Da and contains β-glycosidic bonds, which have been speculated to be major factors explaining why bamboo shoot polysaccharide was not digested by gastric acid and did not promote the proliferation of probiotics. Some studies have shown that, when used as prebiotics, low molecular weight polysaccharides have a better proliferation effect on probiotics. Ramnani et al. (7) studied a low molecular weight polysaccharide (64.64 kDa) from agar that has a significant growth-promoting effect on *Bifidobacterium*. After 48 h of *Bifidobacterium* culture in medium containing this polysaccharide, *Bifidobacterium* numbers increased from 8.06 to 8.55 log CFU. At the same time, the content of short-chain fatty acids increased, especially that of acetic acid and propionic acid, indicating that the polysaccharide can be used by probiotic bacteria. In the differential global transcriptome result, during logarithmic growth of *L. acidophilus* NCFM using GOS or glucose as a sole source of carbohydrate. *lacS*, a galactoside-pentose-hexuro-nide permease-encoding gene, was up-regulated 5.1-fold in the presence of GOS (8). The same kinds of commercial prebiotics or plant prebiotics extracted in laboratories show different degrees of proliferation-promoting effects on different probiotics. In addition, various laboratory-extracted prebiotics or plant prebiotics also show different degrees of proliferation-promoting effects on the growth of the same types of probiotics (5). Therefore, the interaction mechanisms between specific prebiotics and probiotics need to be studied in depth.

*Lactobacillus rhamnosus* GG (LGG) is currently one of the most recognized probiotic strains in the world. LGG can adhere to the intestinal mucosa and epithelial cells, has strong acid resistance, and can survive passage through the gastrointestinal tract. It can colonize the intestines, become a part of the normal intestinal flora, and simultaneously, promote the growth of beneficial bacteria (*Lactobacillus* and *Bifidobacterium*) in the gut (9, 10). LGG can provide many benefits to human health, including inhibiting the growth of harmful bacteria (11), inhibiting the production of harmful enzymes or other harmful substances, preventing respiratory infections (12), preventing allergies (13), preventing or treating diarrhea (14, 15), and improving immunity (16).

In our previous study, mulberry oligosaccharide was produced from mulberry polysaccharides by physical ultrasonic hydrolysis, chemical acid hydrolysis, and enzymatic hydrolysis. Among four probiotic bacteria, the growth of *L. rhamnosus* was affected the most by oligosaccharides produced with β-mannanase hydrolysis (17). The enzymatically prepared mulberry oligosaccharides were also superior to two commercial prebiotics (galactooligosaccharides and isomaltooligosaccharides). The mulberry oligosaccharides were then further purified by using DEAE-52 cellulose and Sephadex G-100 columns. The purified oligosaccharides were collected and freeze-dried for further physical and chemical analysis, which showed that the oligosaccharide consists of galactose and has an average molecular weight of 987 Da. Because the purified oligosaccharides only contain galactose units, we named it mulberry galactooligosaccharide (MGO). The proliferation of *L. rhamnosus* reached 1,420% when 4% (*w/v*) MGO was added, when the number of colonies produced by culture in MRS medium without added oligosaccharide was defined as 100% proliferation (18). Research has shown *Lactobacillus* to be beneficial to the health of the host (19, 20), and MGO can increase the content of *Lactobacillus* in the body. However, the molecular mechanisms underlying LGG proliferation when different kinds of prebiotics are added to MRS (de Man, Rogosa and Sharp) medium are highly complex, and multiple genes, proteins, metabolites, and bio-processes are likely to be involved. Understanding the regulatory pathways and molecular mechanisms of the MGO promotion of LGG proliferation is of great significance for the development of intestinal prebiotic mulberry foods.

In this study, we used LGG as a model strain and cultured it with different prebiotics in MRS medium. We initially used transcriptomics and metabolomics to screen for genes with differential expression. Then, real-time quantitative PCR (qPCR) experiments were employed to examine selected differential genes to verify the accuracy and repeatability of the RNA-seq data. These results provide the foundation for understanding the mechanism by which MGO promotes the proliferation of LGG.

## Materials and Methods

### Chemicals and Strain

MRS medium and agar powder were purchased from Guangdong Huankai Microbial Sci. & Tech. Co., Ltd. (Guangzhou, China). Galactooligosaccharides (GOS) and β-Mannanase (50 U/mg) were purchased from Shanghai Yuanye Bio-Technology Co., Ltd. (China). All other chemicals were of analytical grade and purchased from Guangzhou Chemical Reagent Factory (Guangzhou, China). *Lactobacillus rhamnosus* GG (ATCC 53103) was obtained from a laboratory culture collection 25% glycerol stock maintained at −80°C and propagated in MRS medium with shaking at 37°C.

### Preparation of MGO

Fresh mulberry, obtained from Huadu Bosun field production (Guangzhou, Guangdong, China), was washed with water and dried using a heat pump at 70°C for 5 h to bring humidity below 30%. Briefly, MGO was isolated as previously described (18), and polysaccharides were extracted from mulberry powder (500 g, crushed mulberry and passed through a 50-mesh screen) with water (10 L) at 70°C for 6 h. The extraction was reduced to 1/5 of its original volume by vacuum filtration at 45°C, followed by precipitation for 24 h with four volumes of 95% (*v/v*) ethanol at 4°C. The precipitate was collected by centrifugation (9,000 × *g* for 20 min), and this precipitate (crude mulberry polysaccharide solution, MPS) was incubated with 5% (*w/v*) β-mannanase at 50°C for 4 h and subsequently lyophilized. The crude precipitate (mulberry oligosaccharide solution) was further purified using DEAE-52 cellulose and Sephadex G-100 columns. The purified oligosaccharide was collected and lyophilized for further experiments. Based on previous research results, this purified oligosaccharide consists of galactose and has an average molecular weight of 987 Da (18). Because the purified mulberry oligosaccharide only contains galactose, it was named MGO.

### Effects of Different Prebiotics on the Growth of LGG

LGG was employed to investigate the effects of GOS, MPS, and MGO on bacterial growth, and GOS was used as a positive control. Medium without polysaccharides or oligosaccharides was used for the negative control. GOS, MPS, or MGO was added to MRS (de Man, Rogosa, and Sharpe) medium at a final concentration of 4% (*w/v*). The mixtures medium was then inoculated with 1% (*v/v*) of an overnight culture of LGG and incubated with shaking at 180 rpm at 37°C for 18 h. Samples were plated every 3 h to count the number of resulting colonies.

### RNA-Seq and Real-Time Quantitative PCR Validation

#### RNA Extraction

The initial logarithmic phase (8 h) and stationary phase (24 h) of LGG growth were selected as the sampling time points for comparative transcriptomics. For transcriptome sequencing, cells in MRS medium were separated by centrifugation (9,000 × g, 10 min), washed with PBS three times, and immediately frozen in liquid nitrogen. Total RNA was extracted from the tissue using TRIzol reagent in accordance with the manufacturer's instructions (Invitrogen, Carlsbad, CA, USA), and genomic DNA was removed using DNase I (TaKaRa, Bio Inc., Dalian, China). Then RNA quality was determined with a 2100 Bioanalyser (Agilent Technologies Co., Ltd., Colorado Springs, CO, USA) and quantified using the ND-2000 (NanoDrop Technologies). An RNA-seq transcriptome library was prepared with the TruSeq RNA sample preparation Kit from Illumina (San Diego, CA, USA) using 2 μg of total RNA. Shortly after preparation, ribosomal (r)RNA depletion, instead of poly (A) purification, was performed using the Ribo-Zero Magnetic kit (Epicenter), then all mRNAs were broken into short fragments by adding fragmentation buffer. Double-stranded cDNA was synthesized using a SuperScript double-stranded cDNA synthesis kit (Invitrogen) with random hexamer primers (Illumina). The ligation products were size-selected *via* agarose gel electrophoresis, amplified *via* PCR using Phusion DNA polymerase (NEB) for 15 cycles, and sequenced using Illumina HiSeq × TEN (2 × 150 bp read length).

#### Bioinformatics Analysis and Differential Expression Analysis

The data generated from the Illumina platform were used for bioinformatics analysis. All of the analyses were performed using the free online platform Majorbio Cloud Platform (www.majorbio.com) at Shanghai Majorbio Bio-pharm Technology Co., Ltd. For each data set, and for each alignment and quantification protocol, we identified differentially expressed genes using the edgeR (http://www.bioconductor.org/packages/2.12/bioc/html/edgeR.html), DESeq2 (http://bioconductor.org/packages/release/bioc/html/DESeq2.html), and DESeq (http://www.bioconductor.org/packages/release/bioc/html/DESeq.html) packages.

#### Gene Ontology and Kyoto Encyclopedia of Genes and Genomes Enrichment Analysis

Differentially expressed genes (DEGs) between the control and treatment groups were determined using gene ontology (GO). GO enrichment analysis was used to identify GO terms that are enriched with DEGs and to illustrate the difference between two particular samples at the functional level.

Metabolic pathways analysis was carried out with the Kyoto encyclopedia of genes and genomes (KEGG) database to identify the most important biological metabolic pathways and signal transduction pathways related to the DEGs.

#### Validation of RNA-Seq Results by RT-qPCR

Twelve DEGs associated with galactose metabolism (*gatB, gatC, lacC, galK, galU*, and *galE*) or glycolysis/gluconeogenesis (*bglA, fbaA, pdhA, pdhB, aceF*, and *lpd*) were selected for validation by RT-qPCR to examine the reliability of the RNA-seq results. Total RNA was extracted using a total RNA extractor with a TRIzol kit (Tiangen, Beijing, China), and reverse transcription and RT-qPCR were performed using a PrimeScript RT Reagent Kit (Takara) and the ABI7300 drop digital PCR system (Applied Biosystems, Foster City, CA, USA), respectively, in accordance with the manufacturers' instructions. The gene names and primer sequences used for qRT-PCR are shown in Table 1. The PCR programs included 40 cycles of 95°C for 5 s, annealing at 55°C for 30 s, and extension at 72°C for 40 s. We amplified the 16 SrRNA gene as an internal standard to normalize gene expression. Three independent repetitions of each sample were performed, and the 2^−ΔΔCt^ method was used to calculate the relative expression levels of the genes.

**Table 1:** List of primers for qRT-PCR.

**Gene**	**Forward primer (5'-3')**	**Reverse primer (5'-3')**
LGG_RS12740 (*gatB*)	CCGGTACTTTTGGTTCTTTT	CTTGGGCACTTTTTTCATTC
LGG_RS12745 (*gatC*)	ACGGTGGTTGCGTCTAAGAT	GCAGAGGCAGTCCATGAATA
LGG_RS01630 (*lacC*)	TCAGGCAGAAACACGTAGCT	CAATCCTTGTGGCATAGAAC
LGG_RS03075 (*galK*)	TTGACATTCCGTTCCCTGAT	CATTTTCGACTTTAACCCCG
LGG_RS05080 (*galU*)	GACCATTCAATTTATCGTTG	CGTCTTTATGTTTTTCTTTC
LGG_RS09660 (*galE*)	TGCACAAACGGACAAGTTGG	CTGAACCGACGCCACATACT
LGG_RS05090 (*bglA*)	CGACGGCCCAAACAAAGAAG	CGAGATCGTGACAAGTGGCT
LGG_RS02500 (*fbaA*)	CAGCTACCGGGATCGACTTC	TAAGCGGTCGAAGTGCAGAC
LGG_RS06325 (*pdhA*)	GCCAGCATGATCGGTAGTCA	AGCGGTAACCCGTGTTGAAT
LGG_RS06330 (*pdhB*)	TTGGCGAACGACCCTAAGAC	ACACGATCCTCACCGTGTTT
LGG_RS06335 (*aceF*)	CAGTCGCGCCTTACGTTTCG	CATGCGGGCTCCGTTGTTTC
LGG_RS06340 (*lpd*)	ACCGGTGGCCTAAACTTACC	CCTAAGTTGGCATAGGCGCT
LGG_16SrRNA	AAGGGTTTCGGCTCGTAAAA	TGCACTCAAGTTTCCCAGTT

### Metabonomic Analysis

#### Sample Preparation for Metabolomics

The initial logarithmic phase (8 h) and stationary phase (24 h) of LGG growth were selected as the sampling time points for comparative metabolomics, and six biological replicates were used per sample. Samples stored at −80°C were thawed at room temperature, and the following steps were conducted by Majorbio Bio-Pharm Technology Co., Ltd. To 50 mg of each sample, 400 μL of methanol extraction solution (methanol-water, 4:1, v/v) was added. The mixture was allowed to settle at −10°C and processed with a Wonbio-96c high-throughput tissue crusher (Shanghai Wanbo Biotechnology Co., Ltd.) at 50 Hz for 6 min, followed by 40 kHz ultrasound treatment for 30 min at 5°C. The solution was kept at −20°C for 30 min to precipitate the protein. After centrifugation at 13,000 × *g* at 4°C for 15 min, the supernatant was carefully transferred to sample vials for LC-MS/MS analysis. A pooled quality control (QC) sample was prepared by mixing equal volumes of all samples, and the QC mix was processed and tested in the same manner as the analytic samples. The QC mix was injected at regular intervals (every six sample runs) to monitor the stability of the analysis.

#### LC-MS/MS Metabolomics Equipment and Parameters

LC-MS/MS was performed on an AB Sciex Triple TOF 5600™ mass spectrometer system (AB SCIEX, USA). The following steps and LC conditions were followed by the Majorbio Bio-Pharm Technology Co.: a BEH C18 column (100-mm × 2.1-mm i.d., 1.7 μm; Waters, Milford, USA) was used; the flow rate was set to 0.4 mL/min, and the sample injection volume was 10 μL. The mobile phases consisted of 0.1% formic acid in water:acetonitrile (95:5, v/v) (solvent A) and 0.1% formic acid in acetonitrile:isopropanol:water (47.5:47.5:5, v/v) (solvent B). The solvent gradient changed according to the following conditions: from 0 to 0.5 min, 0 to 0% B; from 0.5 to 2.5 min, 0% B to 25% B; from 2.5 to 9 min, 25% B to 100% B; from 9 to 13 min, 100 to 100% B; from 13 to 13.1 min, 100 to 5% B; and from 13.1 to 16 min, 5 to 5% B for equilibrating the systems. The column temperature was maintained at 40°C. During the analysis, all samples were stored at 4°C.

The UPLC system was coupled to a quadrupole time-of-flight mass spectrometer (Triple TOF 5600+, AB Sciex, USA) equipped with an electrospray ionization source operating in positive mode and negative mode. The optimal conditions were set as follows: source temperature, 550°C; curtain gas, 30 psi; ion source GS1 and GS2, both 50 psi; ion-spray voltage floating, −4,000 V in negative mode and 5,000 V in positive mode; de-clustering potential, 80 V; collision energy, 40 ± 20 V rolling for MS/MS; cycle time, 510 ms. Data acquisition was performed with the data-dependent acquisition mode, and detection was carried out over a mass range of 50–1,000 m/z.

### Statistical Analysis

All data represent the mean ± standard deviation (SD) of at least three replicate measurements, and the results were analyzed on SPSS-19 (Chicago, IL, USA.). Statistical significance (*P*< *0.05*) between treatments was analyzed by one-way analysis of variance, followed by Duncan's multiple-range test. For analysis software, the online platform of Majorbio I-Sanger Cloud platform (https://cloud.majorbio.com/) was used.

## Results and Discussion

### Growth Curve of LGG in MRS Medium With Different Prebiotics

**Figure 1:**
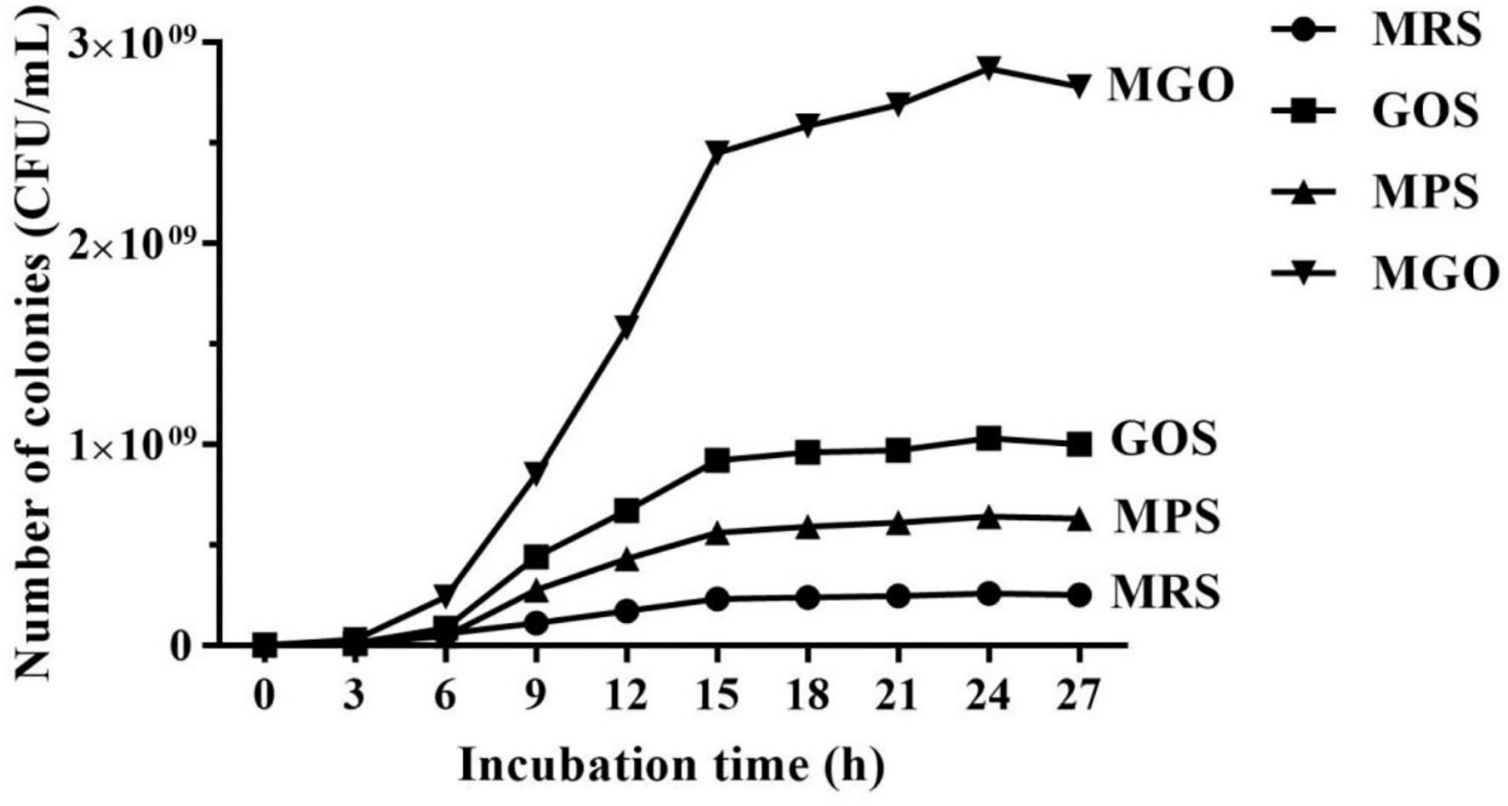
Curve of LGG growth in MRS medium with different prebiotics. MRS, no additional prebiotics added to MRS medium; GOS, 4% (*w/v*) galacto-oligosaccharides added to MRS medium; MPS, 4% (*w/v*) mulberry polysaccharide added to MRS medium; MGO, 4% (*w/v*) mulberry galacto-oligosaccharide added to MRS medium.

The growth curve in Figure 1 shows the effect on LGG when 4% (*w/v*) GOS, MPS, or MGO was added to the MRS medium. After 24 h of culture, the culture reached about 2.9 × 10^9^ CFU/mL when 4% (*w/v*) of MGO was added. The CFU increased about by 3 to 11 times in MGO medium compared with in the GOS (1 × 10^9^ CFU/mL), MPS (6.4 × 10^8^ CFU/mL), and control (2.6 × 10^8^ CFU/mL) media.

### Analysis of Differential Gene Expression

#### Cluster Analysis of Differential Gene Expression

**Figure 2:**
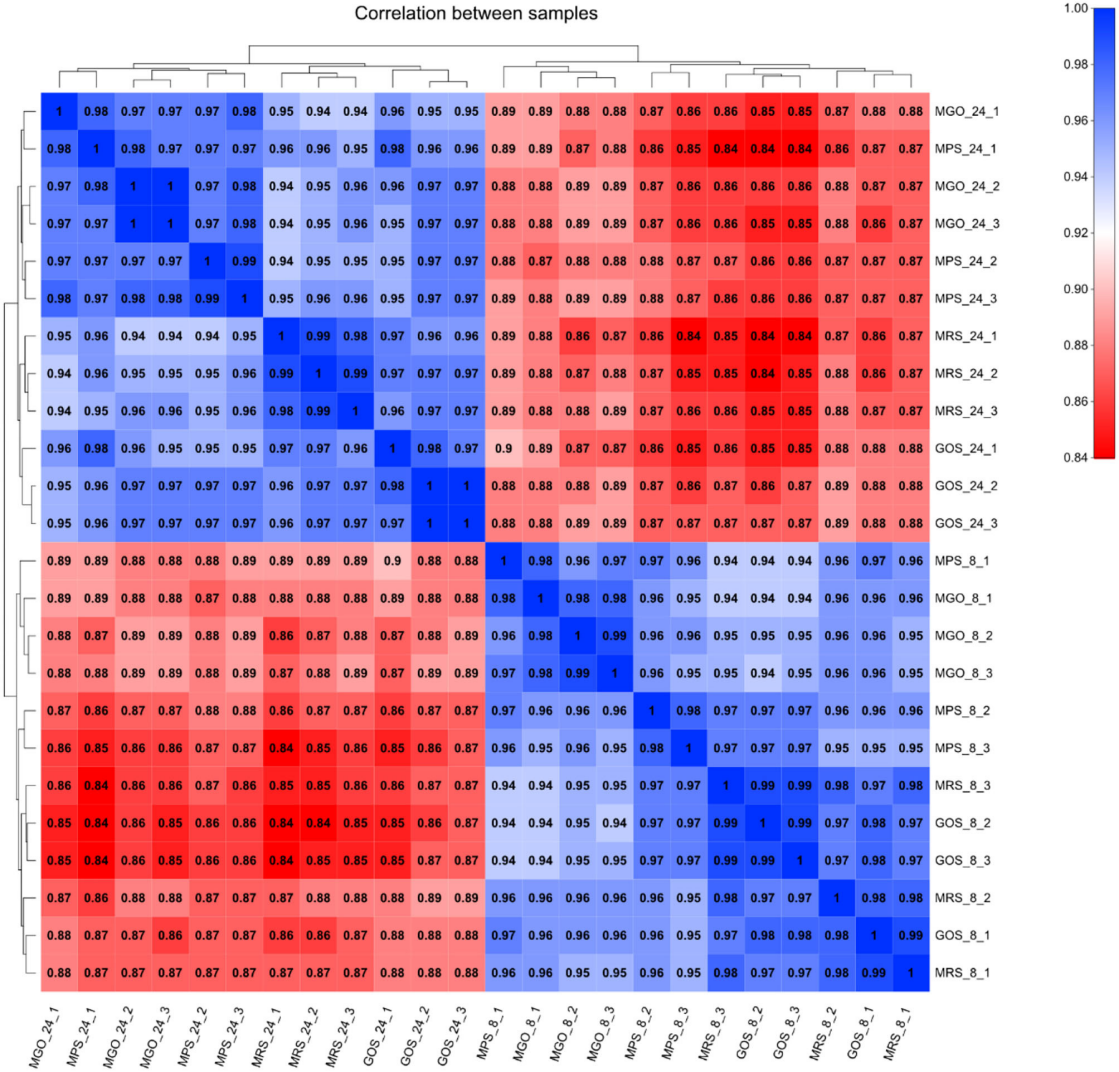
Clustering analysis of differentially expressed genes with different prebiotics.

The reproducibility and significance of gene expression differences between samples can be seen from the Pearson correlation coefficient graph (Figure 2). The higher the intensity of the blue color indicates a higher correlation between two samples and a smaller difference between them. The higher the intensity of the red color indicates the poorer the correlation between two samples and the greater the difference. The number represents the Pearson correlation coefficient between the samples. As illustrated in Figure 2, the biological replicate samples from each test group had good parallelism, and the 8-h and 24-h transcription levels were quite different between the sample groups.

#### Identification of Differentially Expressed Genes

**Figure 3:**
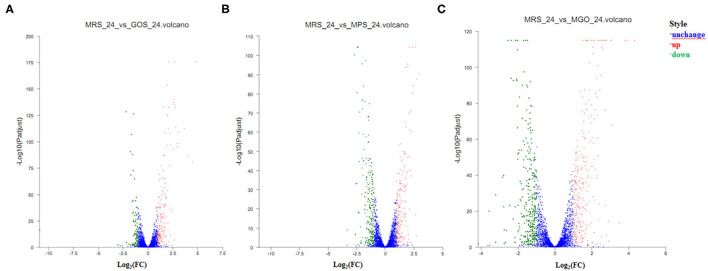
Volcano plots of differentially expressed genes after different prebiotic treatments. Genes with differences in expression are shown in red (up-regulated) or green (down-regulated), and genes with no significant differential expression are shown in blue. The horizontal axis represents the change in gene expression in different samples. **(A)** GOS added to the MRS medium and culture for 24 h; **(B)** MPS added to the MRS medium and culture for 24 h; **(C)** MGO added to the MRS medium and culture for 24 h.

To understand the gene expression responses of LGG to different prebiotic treatments, transcriptome analysis was conducted to identify the DEGs at the 24 h culture stage. GOS, MPS, and MGO were, respectively added to the MRS medium, and after 24 h culture, 179, 212, and 298 genes, respectively, were discovered to be up-regulated as the growth rate increased. Furthermore, 88, 223, and 321 genes, respectively, were shown to be down-regulated as the growth rate increased. The remaining 2876, 2708, and 2524 genes in the respective media did not change with growth rate. The statistical results of the gene quantification are shown in the volcano plot (Figure 3). There were ~63 and 132% more genes differentially expressed when MPS and MGO were added to the MRS medium, respectively, than GOS, of which there were approximately 18% and 66% more up-regulated genes. In terms of promoting the proliferation of probiotics, the proliferation rate of LGG was also higher with MGO treatment than GOS treatment. In the LGG cultivation, slightly more up-regulated genes were seen with MGO than MPS. This may be because prebiotics with a smaller molecular weight are more easily used by LGG. MGO containing six galactose units resulted in a better LGG proliferation rate and stimulated the up-regulation of more genes.

As illustrated in Table 2, when GOS, MPS, or MGO was added to the MRS medium, 17, 10, and 29 genes, respectively, were up-regulated, with a more than 5 log2-fold change. *LGG_RS01420, LGG_RS03150, LGG_RS02910, LGG_RS03140* and *LGG_RS03145* genes, which encode hypothetical protein, DeoR/GlpR transcriptional regulator, amino acid permease, galactose-6-phosphate isomerase subunit LacB and galactose-6-phosphate isomerase subunit LacA, respectively, were highly expressed. The DeoR/GlpR transcriptional regulator is involved in the production of amino acids (including L-lysine) (21, 22), and amino acid permease plays an important role in the absorption and transportation of amino acids (23, 24). Galactose-6-phosphate isomerase can catalyze D-6-phosphate-galactose to D-6-phosphate-tagatose and participate in the metabolism of galactose (25).

**Table 2:** Differentially expressed genes (fold change >5) of LGG cultured for 24 h in different prebiotic media.

**Gene_id**	**Gene description**	**GOS/MRS**	**MPS/MRS**	**MGO/MRS**
LGG_RS00640	MurR/RpiR family transcriptional regulator	5.981	6.188	13.73
LGG_RS00645	TetR/AcrR family transcriptional regulator	6.539	6.673	15.04
LGG_RS01415	Branched-chain amino acid transport system II carrier protein		5.017	8.158
LGG_RS01420	Hypothetical protein	29.647		
LGG_RS01495	ABC transporter substrate-binding protein			5.408
LGG_RS01785	LacI family DNA-binding transcriptional regulator	6.934		
LGG_RS02525	Dipeptide epimerase			5.831
LGG_RS02720	Amino acid ABC transporter permease			6.044
LGG_RS02730	Branched-chain amino acid transport system II carrier protein			5.6
LGG_RS02895	ABC-F type ribosomal protection protein		5.299	8.473
LGG_RS02910	Amino acid permease		8.007	19.921
LGG_RS03135	Tagatose-bisphosphate aldolase	8.159		
LGG_RS03140	Galactose-6-phosphate isomerase subunit LacB	12.353		
LGG_RS03145	Galactose-6-phosphate isomerase subunit LacA	16.671		
LGG_RS03150	DeoR/GlpR transcriptional regulator	22.369		
LGG_RS04110	NUDIX hydrolase		5.226	
LGG_RS06015	Amino acid permease			6.305
LGG_RS08675	Copper-translocating P-type ATPase		5.179	5.754
LGG_RS10195	Nitronate monooxygenase	5.405		
LGG_RS10200	Acyl carrier protein	6.225		
LGG_RS10205	Ketoacyl-ACP synthase III	5.945		
LGG_RS10210	MarR family transcriptional regulator	6.707		
LGG_RS10215	Beta-hydroxyacyl-ACP dehydratase	7.904		
LGG_RS10325	Proline/glycine betaine ABC transporter permease			6.358
LGG_RS10330	Glycine betaine/L-proline ABC transporter ATP-binding protein			5.21
LGG_RS10520	PTS glucose transporter subunit IIA	6.401		
LGG_RS10665	Hydrolase	8.037		
LGG_RS11005	Nucleoside 2-deoxyribosyltransferase			5.071
LGG_RS11410	Threonine/serine exporter family protein			6.95
LGG_RS11560	Hypothetical protein			5.5
LGG_RS11590	30S ribosomal protein S14			5.793
LGG_RS12095	NAD(P)H-dependent oxidoreductase			5.172
LGG_RS12585	DUF1275 domain-containing protein			6.815
LGG_RS14005	Carbon-nitrogen family hydrolase	6.421	6.575	11.35
LGG_RS14010	MetQ/NlpA family ABC transporter substrate-binding protein			7.613
LGG_RS14065	tRNA uridine-5-carboxymethylaminomethyl(34) synthesis GTPase MnmE			5.659
LGG_RS15770	Putative metal homeostasis protein			6.979
LGG_RS16050	D-ribose pyranase	5.338		
LGG_RS16085	Hypothetical protein		5.009	
sRNA0007				6.649
sRNA0038			6.316	
sRNA0038				5.268
sRNA0053				6.593
sRNA0222				5.053
sRNA0304				7.463
sRNA0318				8.539

### GO Annotation Analysis of DEGs

**Figure 4:**
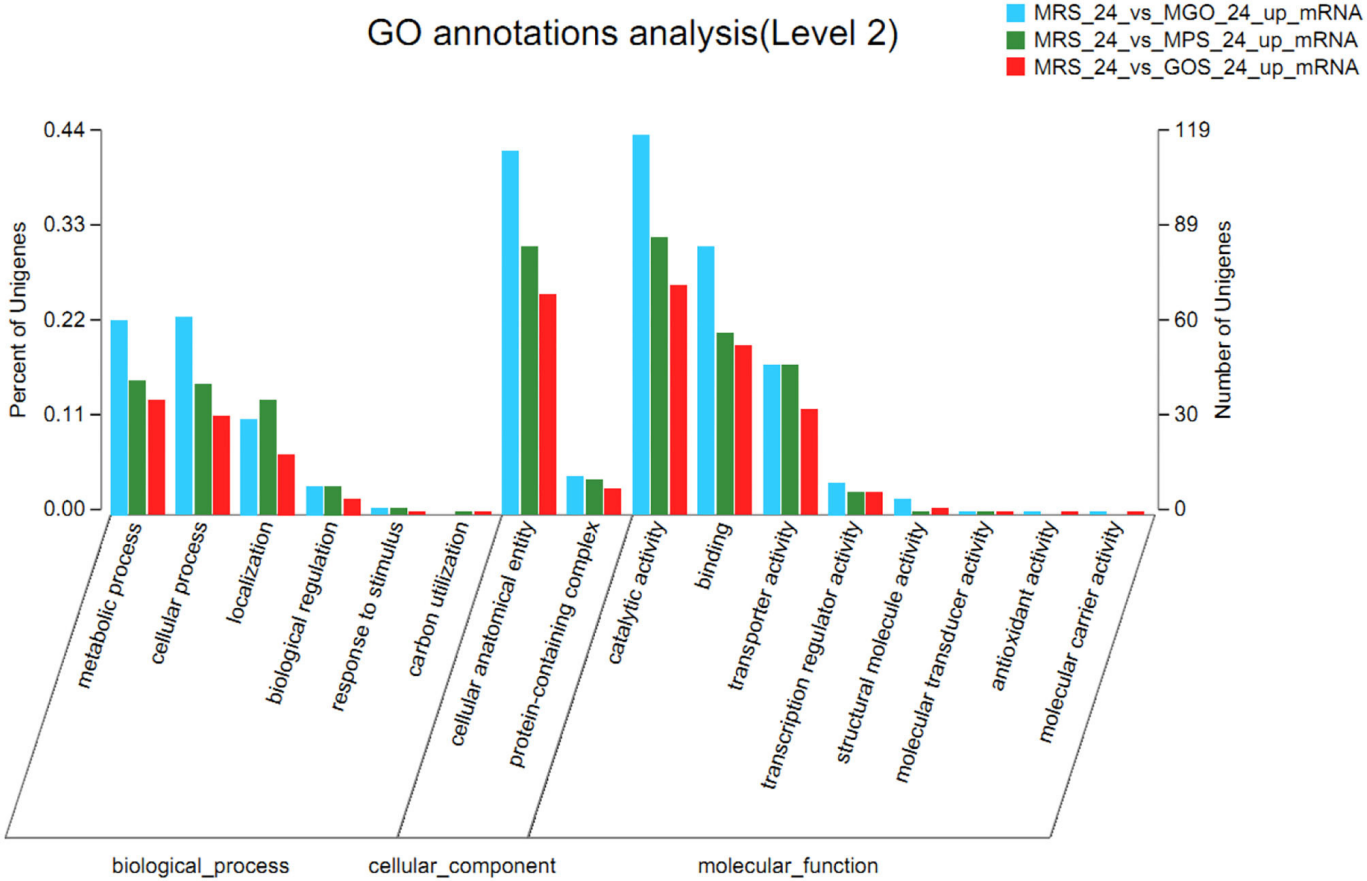
Comparative transcriptomic analysis of LGG cultured with different prebiotics for 24 h. Histogram presentation of differentially expressed gene ontology classifications. The results are summarized in three main categories: biological process, cellular component, and molecular function. The left y-axis represents the percentage of unigenes of a specific category within the main category, and the right y-axis represents the number of unigenes of a specific category within the main category. The bar indicates the number of up-regulated genes.

To clarify the changes in LGG biological processes that occurred after different prebiotics were added, the up-regulated and down-regulated genes identified *via* RNA-seq were subjected to GO term enrichment analysis. As illustrated in Figure 4, up-regulated GO functional gene enrichment analysis resulted in a list of biological processes, cellular components, and molecular functions. The molecular function category contained the majority of the GO annotations, followed by the biological process and cellular component categories. In the ontology of molecular function, the main categories were catalytic activity, binding, and transporter activity. In the biological process ontology, most DEGs were enriched in metabolic processes, cellular processes, and localization. For the cellular component ontology, most DEGs were associated with integral components of cellular anatomical entity and protein-containing complexes. The up-regulated GO functional gene component category for MGO, MPS, and GOS treatments contained 558, 426, and 340 genes, respectively. The highest number of up-regulated GO functional genes was observed when MGO was added to the LGG cultivation.

### KEGG Analysis of DEGs

**Figure 5:**
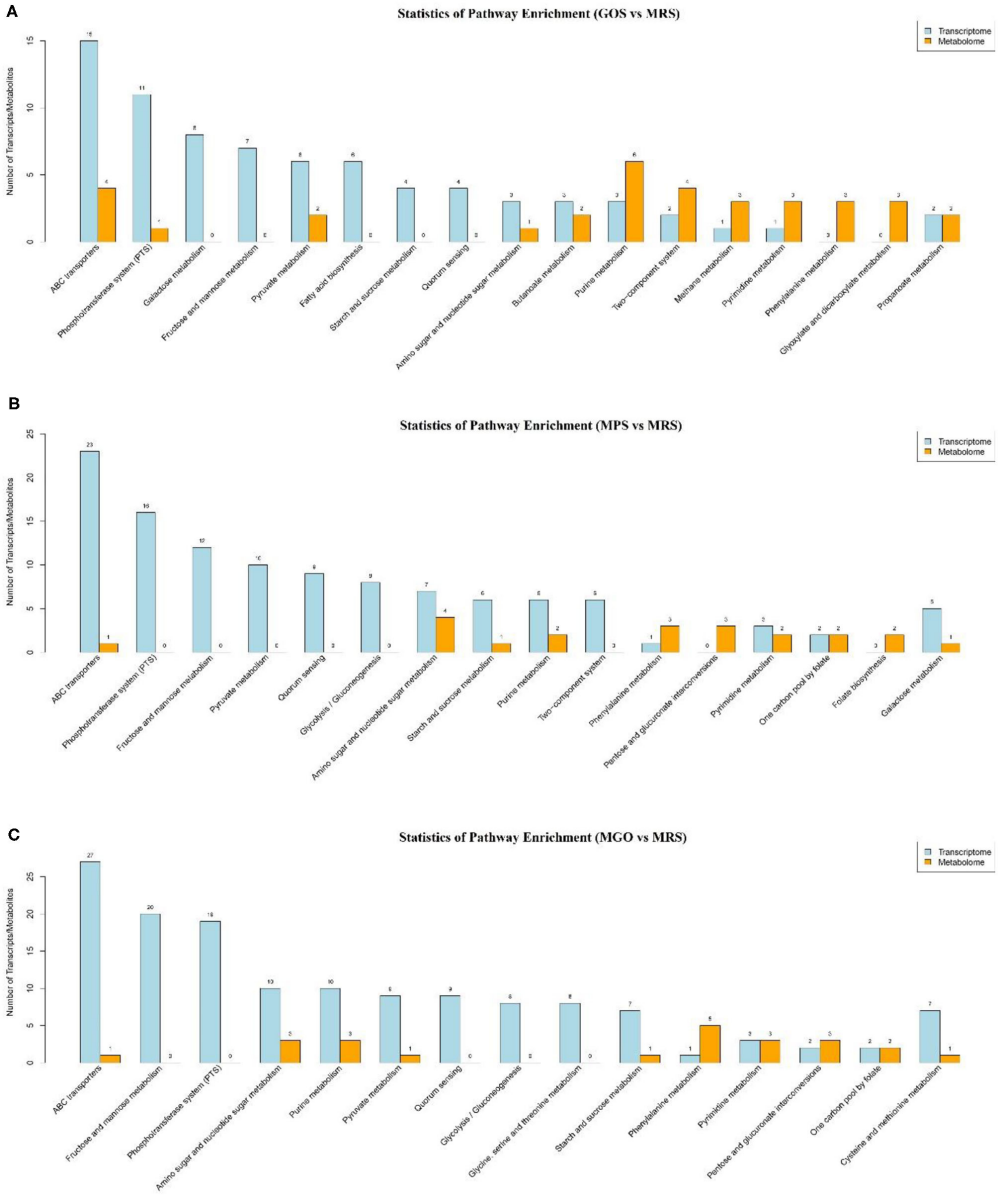
Comparative transcriptomic and metabolomic analyses of LGG cultured with different prebiotics for 24 h. KEGG pathway enrichment analysis of DEGs. The horizontal coordinates represent the KEGG pathway name, and the vertical coordinates represent the number of transcript/metabolite unigenes. **(A)** GOS added to the MRS medium and culture for 24 h; **(B)** MPS added to the MRS medium and culture for 24 h; **(C)** MGO added to the MRS medium and culture for 24 h.

**Figure 6:**
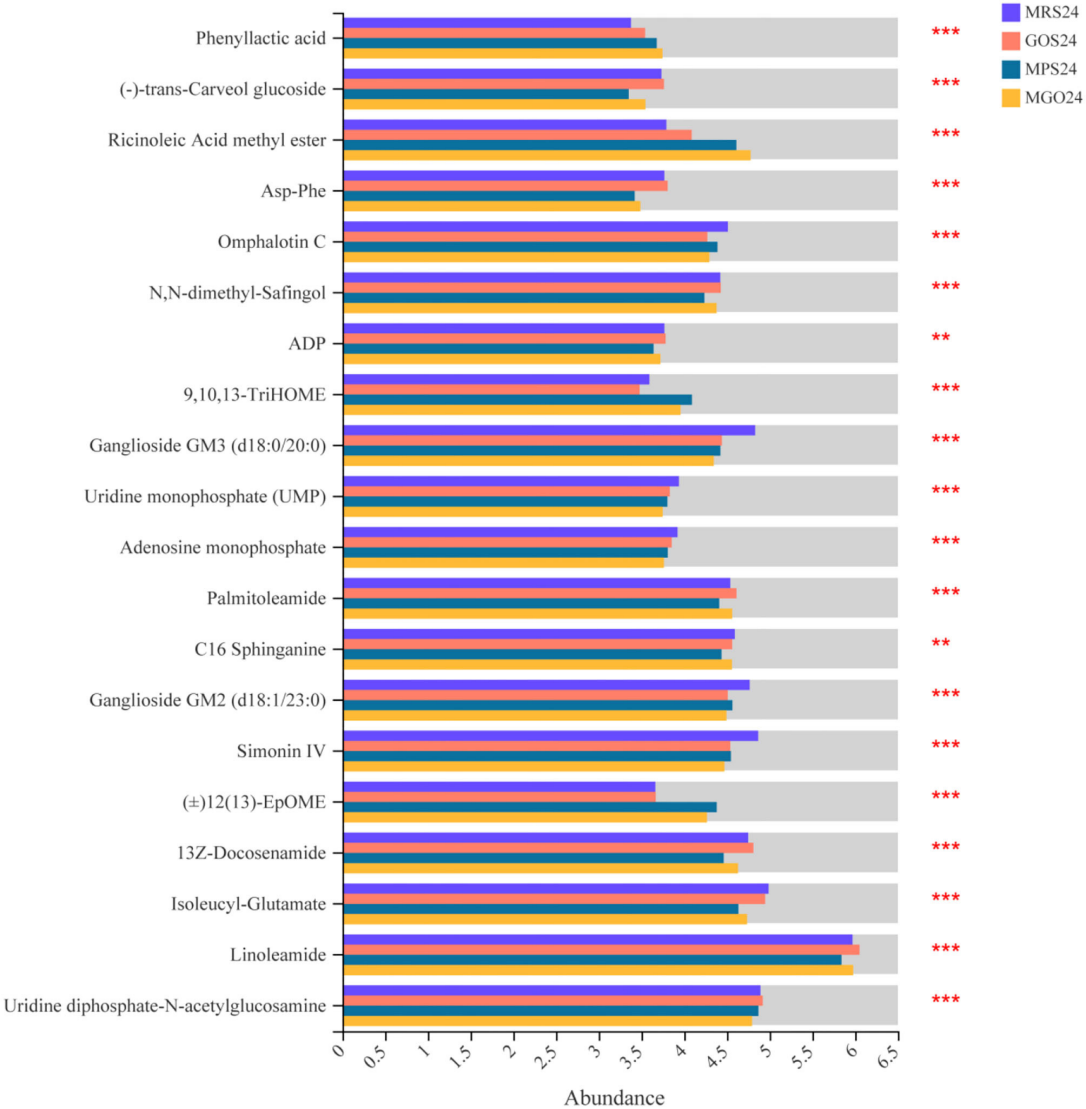
Comparative differential metabolites analyses of LGG cultured with different prebiotics for 24 h. The horizontal coordinates represent the average relative abundance of metabolites in different groups, and the vertical coordinates represent metabolites. **0.001 < *P* ≤ *0.01*, ***P ≤ *0.001*.

To further identify changes in biochemical pathways during treatment with the different prebiotics, all DEGs were further mapped using the KEGG database (26). The top 10 KEGG pathways with the largest number of differential genes and differential metabolites were identified for analysis (Figure 5). Of these, ABC transporters, the phosphotransferase system (PTS), galactose metabolism, fructose and mannose metabolism, pyruvate metabolism, quorum sensing, amino sugar and nucleotide sugar metabolism, purine metabolism, and glycolysis/gluconeogenesis and etc. were enriched. Among these, the five pathways with the largest number of differential genes or differential metabolites when MGO was added to the MRS medium were ABC transporters, fructose and mannose metabolism, PTS, amino sugar and nucleotide sugar metabolism, and purine metabolism. The differences of metabolites were analyzed, and 18 compounds such as Linoleamide, Uridine diphosphate-N-acetylglucosamine, Isoleucyl-Glutamate, 13Z-Docosenamide and Simonin IV were found to be significantly different (Figure 6, *p* ≤ *0.001*). Linoleamide is one of the endogenous fatty acid primary amides and the most of the significantly different metabolites. It was shown that linoleamide appears to be long-chain bases structurally related to sphingosine and sphinganine into which a second unsaturated bond has been introduced (27). Uridine diphosphate-N-acetylglucosamine is an acetylated aminosugar nucleotide. A previous study show uridine diphosphate-N-acetylglucosamine serves as the donor sugar nucleotide for lipid and secretory protein complex glycosylation, glycosyl phosphatidylinositol anchor synthesis, and N-acetylglucosmine modification of nuclear and cytosolic proteins (28). Isoleucyl-Glutamate is a dipeptide composed of isoleucine and glutamate. It is an incomplete breakdown product of protein digestion or protein catabolism. This dipeptide has not yet been identified in human tissues or biofluids and so it is classified as an “Expected” metabolite (29). MGO is an oligosaccharide consisting of galactose, based on the results of the KEGG metabolic pathology analysis, we found UDP-glucose was more abundant in the MGO group than other groups, which may be a key metabolite for MGO to promote LGG proliferation. The abundance of UDP-glucose in MGO group (3.8816 ± 0.0215) was 106, 131 and 137% higher than that of MPS group (3.6770 ± 0.0296), GOS group (2.9741 ± 0.0395) and MRS group (2.8367 ± 0.1465), respectively. UDP-glucose is a nucleotide sugar. It involves the reactions of glycosyltransferases in metabolism. It is a precursor of glycogen, which can be converted into galactose and UDP-glucuronic acid, which are then enzymatically used to make polysaccharides containing galactose and glucuronic acid (30).

**Figure 7:**
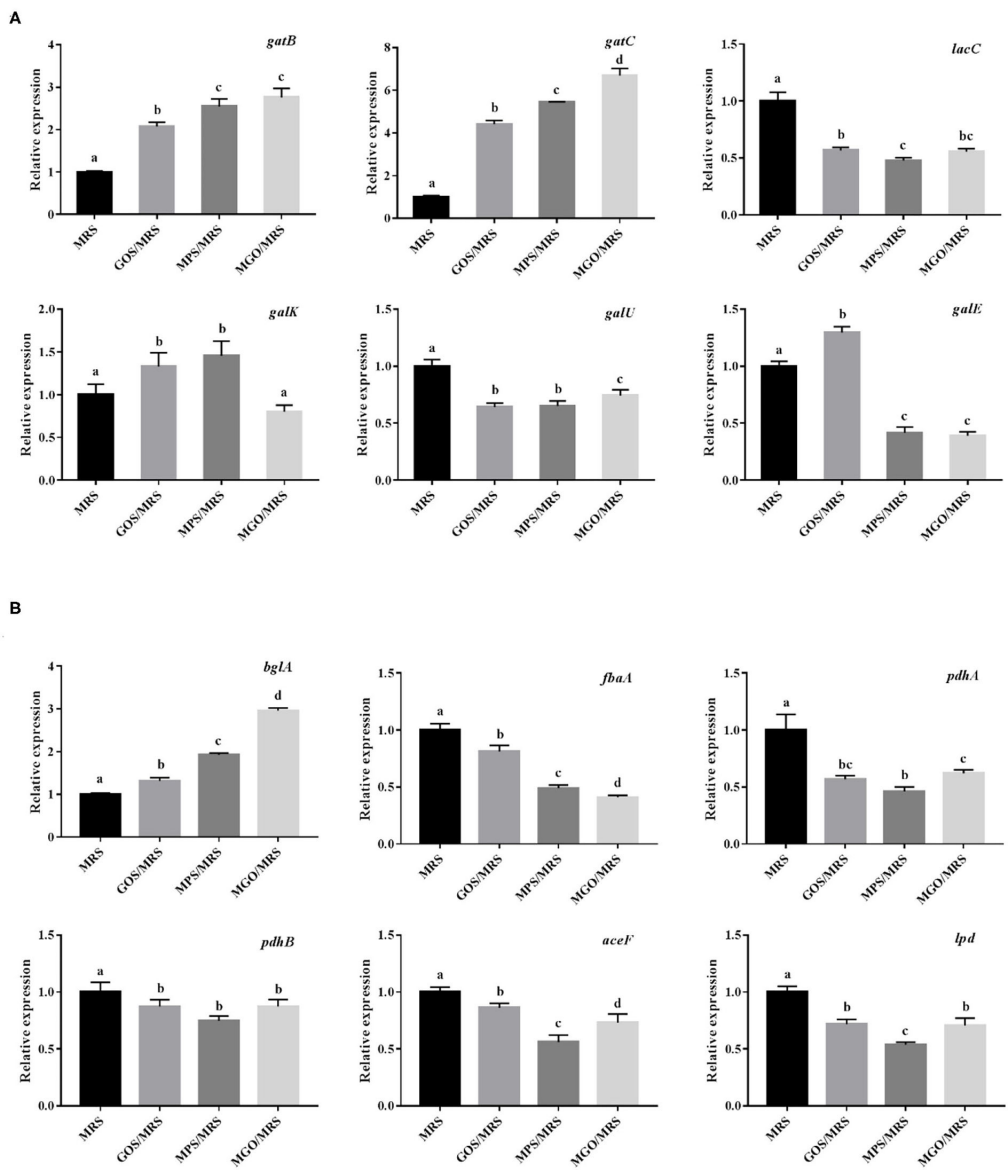
Effects of different prebiotics on the transcription of galactose metabolism pathway-related genes **(A)** and glycolysis/gluconeogenesis pathway-related genes **(B)** in LGG cultured for 24 h, based on RNA-seq data.

Because MGO is an oligosaccharide consisting of galactose, combined with the results of the KEGG metabolic pathology analysis, we speculated the mechanism of MGO proliferation of LGG was the stimulation of genetic changes related to carbohydrate metabolic pathways, specifically galactose metabolism and glycolysis/gluconeogenesis, which promoted important signal pathways for LGG proliferation. To understand the molecular mechanism of the action of MGO on the synthesis of galactose metabolism and glycolysis/gluconeogenesis, we screened for representative DEGs associated with galactose metabolism and glycolysis/gluconeogenesis synthesis in the transcriptomic sequencing results (Table 3). Two DEGs (*gatB* and *gatC*) involved in galactose metabolism were up-regulated, and four DEGs (*lacC, galK, galU* and *galE*) were down-regulated. As shown in Figure 7A, the qRT-PCR data showed the two highest gene expression promotion rates, with an increase of up to 669% (*gatC*) and 276% (*gatB*), when 4% (*w/v*) MGO was added to the MRS medium, which is consistent with the gene expression results obtained from the RNA-seq, suggesting the RNA-seq data were reliable.

**Table 3:** Effect of different prebiotics on galactose metabolism- and glycolysis/gluconeogenesis- related gene transcription in LGG cultured for 24 h.

**Gene ID (KEGG Name)**	**Gene description**	**FC**			**Style**
		**GOS/MRS**	**MPS/MRS**	**MGO/MRS**	
**Galactose metabolism**
LGG_RS12740 (*gatB*)	PTS galactitol transporter subunit IIC	2.267	2.782	2.204	Up
LGG_RS12745 (*gatC*)	PTS sugar transporter subunit IIB	2.551	3.244	2.776	Up
LGG_RS01630 (*lacC*)	Hexose kinase	0.705	0.433	0.392	Down
LGG_RS03075 (*galK*)	Galactokinase	1.465	1.460	0.492	Down
LGG_RS05080 (*galU*)	UTP–glucose-1-phosphate uridylyltransferase GalU	0.610	0.548	0.464	Down
LGG_RS09660 (*galE*)	NAD-dependent epimerase/dehydratase family protein	1.197	0.331	0.298	Down
**Glycolysis/gluconeogenesis**
LGG_RS05090 (*bglA*)	Glycoside hydrolase family 1 protein	1.183	1.757	2.701	Up
LGG_RS02500 (*fbaA*)	Class II fructose-1%2C6-bisphosphate aldolase	0.952	0.455	0.387	Down
LGG_RS06325 (*pdhA*)	Pyruvate dehydrogenase (acetyl-transferring) E1 component subunit alpha	0.697	0.466	0.454	Down
LGG_RS06330 (*pdhB*)	Alpha-ketoacid dehydrogenase subunit beta	0.677	0.448	0.469	Down
LGG_RS06335 (*aceF*)	2-oxo acid dehydrogenase subunit E2	0.651	0.415	0.427	Down
LGG_RS06340 (*lpd*)	Dihydrolipoyl dehydrogenase	0.606	0.362	0.384	Down

The definitions for *gatB* and *gatC* in the KEGG pathway are galactitol PTS system EIIB component and galactitol PTS system EIIC component, respectively. The bacterial PTS is widely found in bacteria, fungi, and some archaea and is composed of phosphotransferases, such as enzyme I (EI), histidine phosphate carrier protein (HPr or NPr), and enzyme II complex (31). PTS is mainly responsible for the absorption of carbohydrates, such as hexose, 6-deoxyhexose, amino sugar, N-acetyl amino sugar, gluconic acid, pentitol, ascorbic acid, and disaccharides, during energy transportation, and it catalyzes their conversion to their respective phosphate esters (32). PTS is both a sugar transport system that mediates the uptake and phosphorylation of carbohydrates and a very powerful regulator. It participates in the metabolism of carbon and nitrogen sources, regulates the homeostasis of iron and potassium, regulates the virulence of certain pathogens, and mediates stress responses. During these different regulatory processes, the regulatory processes signal is provided by the phosphorylation state of the PTS components, which changes according to the availability of the PTS substrate and the metabolic state of the cell (33, 34).

Only one DEG (*bglA*) involved in glycolysis/gluconeogenesis was up-regulated, and five DEGs (*fbaA, pdhA, pdhB, aceF*, and *lpd*) were down-regulated when 4% (*w/v*) MGO was added to the MRS medium (Figure 7B). In addition, these DEGs were verified by qRT-PCR, which showed that *bglA* expression was promoted by 296% and that of *fbaA, pdhA, pdhB, aceF*, and *lpd* was reduced to 40.9, 61.9, 87.3, 73.0, and 70.6% when LGG was grown in 4% (*w/v*) MGO in MRS medium. The KEGG pathway definition of *bglA* is 6-phospho-beta-glucosidase; this intracellular enzyme of microorganisms catalyzes the hydrolysis of β(1,4)-linked cellobiose to produce glucose and glucose-6-phosphate. Both of these reaction products can further enter the glycolysis pathway to be used in energy production (35, 36). PET-PTS, a phosphoenolpyruvate-dependent sugar PTS, is a multi-component sugar transport system that usually coexists with 6-phospho-beta-glucosidase in cellulose-degrading bacteria. PET-PTS phosphorylates the C6 position of β-glucoside compounds while transporting them into the cell, allowing the phosphorylated products to be decomposed and used by 6-phospho-β-glucosidase (37–39).

## Conclusion

In the present study, we discovered that MGO has a stronger proliferative effect on LGG than GOS and MPS. The transcriptome results showed that after adding MGO to the MRS medium, GO functional analysis showed that 558 genes were up-regulated as the LGG growth rate increased, and these genes were mainly enriched for cellular processes, cellular anatomical entities, and catalytic activity. When differential genes were mapped to the KEGG database, we found that genes that were up-regulated with the increasing growth rate were mainly enriched in the membrane transport, amino acid metabolism, and carbohydrate metabolism pathways. Of the significantly up-regulated genes, *gatB* and *gatC* are related to galactose metabolism, and *bglA* is related to the glycolysis/gluconeogenesis pathway. The metabolomics results showed UDP-glucose may be a key metabolite for MGO to promote LGG proliferation.

## Data Availability Statement

The datasets presented in this study can be found in online repositories. The name of the repository and accession number can be found at: NCBI; PRJNA800608.

## Author Contributions

EL: methodology, software, funding acquisition, and writing—original draft. QZ: investigation and formal analysis. DP: methodology. FL: formal analysis. SL: funding acquisition. YZ: project administration. All authors contributed to the article and approved the submitted version.

## Funding

This work was supported by the National Natural Science Foundation of China Grant (No. 31801525), China Agriculture Research System of MOF and MARA, Natural Science Foundation of Guangdong Province (2018A030313874), Agricultural Competitive Industry Discipline Team Building Project of Guangdong Academy of Agricultural Sciences (No. 202119TD), Guangdong Modern Agricultural Industry Technology System Innovation Team (2021KJ124), and Science and Technology Projects of Guangzhou City (202102020965).

## Conflict of Interest

The authors declare that the research was conducted in the absence of any commercial or financial relationships that could be construed as a potential conflict of interest.

## Publisher's Note

All claims expressed in this article are solely those of the authors and do not necessarily represent those of their affiliated organizations, or those of the publisher, the editors and the reviewers. Any product that may be evaluated in this article, or claim that may be made by its manufacturer, is not guaranteed or endorsed by the publisher.
